# Infantile Therapeutics

**Published:** 1856-08

**Authors:** 


**THERAPEUTIC RESULTS IN THE CHILDREN'S HOSPITAL OF MUNICH,**

UNDER PROFESSOR HAUNER.

Reported in the Journal f. Kinderkrankheiten ; 1855. Translated from l'Union Medicale for March, 1856 : by M. Morton Dowler, M. D., New Orleans.

The distinguished Bavarian Professor has briefly recited his own observations touching the action of medicines by him employed in diseases of children. His judgment is of great value, not only from his eminent ability, but from the fact that the hospital of which he is at the head, receives annually more than 2,000 sick children; and during many years he has occupied the post of chief physician.

He sets out by recommending *vaccination* in cases of *nævus maternus*; and recommends the inoculations to be performed not only all over the surface of the *nævus*, but even on the sound skin on the margin of the part affected.

1. *Chlorate of Potassa.*—Employed for many years with constant success in *stomatitis* and ulcers of the *mouth*, and in more than seventy cases. The results are wonderful; for in four hours the penetrating, disagreeable odor of the mouth disappears, and the cure is rapid. It is very valuable in diphtheritic affections of the mouth and throat, and in mercurial ulcerations ; but its value is less constant in these cases:—℞. *Potas. Chlorat.*, ℈ss–℈j ; *Aq. Distillat.*, ℥iij–℥jv ; *Syrup Alb.* ℥ss ; *m. ft. mist. S.* To be used in 24 hours.

[We have found the Chlorate Potassa a most valuable remedy in ulceration of the mouth in ptyalism, in stomatitis materna, and also internally in cases of mercurialization in excess. In these cases we have used it both as a wash and internally, with the most gratifying results.—Eds. Journal.]

2. *Cod Liver Oil.*—Has been given in at least 200 cases of rickets, of every form; all of which have been cured. The other hygienic and medicinal means, as baths, lotions, etc., have not been the principal curative means; for, employed without the oil, the cure has not been effected. The oil is given in doses of from three to four dessert spoonfulls a day. Gastric and diarrhœal affections, if existing, must be treated before giving the oil. In summer, and during warm days, it is much less easily supported than in winter, and in cool weather.

3. *Tincture of Artificial Musk.* (*Musc Ambree.*)—In laryngeal spasm, uncomplicated. Nothing is said of either dose or formula. Employed with constant success in more than thirty cases.

4. *Arsenic.*—*Fowler's Solution*, in various cutaneous eruptions; as the different forms of eczema, prurigo, psoriasis guttata, &c. The constitutional origin of these affections, is first sought out, and the treatment governed by the same. Those which resist the ordinary rational and empirical treatment, are amenable to arsenic. ℞. *Liq Potas. Arsenical.*, ʒj; Aq. Destill, ʒss; S. Take five drops, at first twice, and afterwards three times a day. At the expiration of one month, the Fowler may be given pure. Fatty and impure aliments must be avoided, and the patient must be warmly clad. Baths hasten the cure. No serious result has been observed from the medicine, but the existence of diarrhœa, either contra-indicates the use of the agent, or neutralizes the result.

[We have combined the above with the decoction of dulcamara in cutaneous diseases, and were well pleased with the procedure.—Eds. Journal.]

5. *Nitrate of Silver* in the decline of *croup*, rubeolic cough, *inflammatory angina*, and in ophthalmia of the new-born. (*Abortive Treatment.*)

6. *Sulphuric Acid, prepared*; *Elixer of Haller.*—This medicine, so readily taken by patients of all ages, has rendered the greatest service in typhoid, and exanathematous fevers; in diseases of the blood and of the vascular system, appertaining to scorbutic affections, especially in the *Spotted Disease of Werlhoff.* It is given with other appropriate remedies, by adding from ʒss. to ʒj. of the medicine, to from ℥jss to ℥jvss. of the syrup of marsh-mallows, and adding a table-spoonful of this to the common drinks.

7. *Arnica.*—M. Hauner having witnessed the abuse of this remedy at the beginning of his career, completely laid it aside. Nevertheless, of late, he has used it on the recommendation of Dr. Schneider in three cases ot *serous exudation*, in children notably scrofulous, but free from any specific dyscrasia. The first two were children brought to the hospital with pleuritic effusions, which had been misunderstood, having been regarded by Dr. S. as purulent. The debility was so great that thoracentesis could not be ventured upon. The ordinary treatment was unavailing. A complete cure was effected by arnica. M. Hauner obtained similar results in the city, in case of a woman of twenty years of age. The third child was attacked with an arachnoidian effusion, with well marked symptoms; and which slowly disappeared.

In all these cases perseverance is requisite; for those affections do not rapidly yield. These four cases do not afford sufficient data on which to determine the value of arnica; but they afford new exemplifications of its value. Take of flowers of anica ʒss–ʒj, or ʒjss, to make from ℥ij to ℥ijss. of infusion; to which ℥ss of Syrup of Senega is to be added; and one or two dessert-spoonsful are to be taken every two hours. The woman took ℥ss of the flowers.

8. *Chinchona*, *Quinine*.—In intermittent fevers, used both internally and endermically. There exists yet another from of fever, nearly approaching typhoid, characterized by feebleness at the onset; and which calls for the employment of quinine. This is the *stupid typhoid fever* of the ancients; the *remittent fever of children*, *without localization*, of many authors. The diagnosis of the disease is very difficult. It is wanting in the usual dry and red tongue, the febrile pulse, the abdominal sensibility, especially the pain in the ilio-cæcal region, the distension of the belly, typhoid stools, etc.; indeed, almost all the signs which characterize typhoid. It is not a cerebral affection; for the history, the primary spmptoms, the condition of the eyes, the absence of vomiting, the state of the abdomen, the position of the patient, the intervals of relief, all show the contrary. This disease is no more nor less than a peculiar species of nervous excitation in excitable children, which is most frequently found during the years of evolution. If unfortunately we have employed antiphlogistics, or purgatives, or if we have refrained from treatment, there results a long continued disease, wasting the patient with hectic fever; or, indeed, the disease may localtze itself on the brain or lungs, according to the individual predisposition of the patient; and carry off the child by a secondary affection. Quinine is the true panacea against this fever.

Quinine has the most salutary influence in hectic fever, even when owing to the existence of tubercles. M. Hauner cites cases of six girls, from six to ten years of age, consumptives, situated under unfavorable circumstances, in which the cough, sweats, emaciation, (and pulmonary symptoms?) which had resisted other means, disappeared under the use of quinine. If quinine rarely cures such cases, it often gives relief.

Many of the neuroses, and sporadic affections, are also cured by quinine.

In *anæmia* of children, and *atrophy* from bad diet, etc., without localization or disorganization of an important organ, the extract of chinchona, cold, prepared by maceration, renders the greatest service:—℞. *Ext. Chinchonæ*, gr. jvss–viij; *Aq. Flor. Anthemidis*, ℥x–℥ij; *Syrup Cort. Aurant.*, ℥ss; *S. Two dessert spoonfuls every two or three hours.*

[We are in the habit of prescribing the chinchona and quinine as a succedanum in the treatment of intermittents with the view of preventing its return. After the regular paroxysms have been broken up we advise the following:—℞. *Ext. Chinchonæ*,

ʒj; *ext. humulus lup*, ʒss,; *ext. taraxucum*, ℈ij; *aromatic sulph. acid*, f ʒss; *sulph. quinine*, gr. x; *aqua distil*, f ℥vj. M. Liq. *One to two teaspoonfuls three times a day.*—Eds. Journal.]

9, 10. *Chamomile*, and *White Decoction.*—The first is an especial manner relieves colic brought about by an accumulation of gas in the intestines, and it acts as a carminative. The second is a pure mucilaginous substance, indicated in case of bronchial and pulmonary irritation, from what cause soever it may originate. Chamomile is generally employed in *baths*, containing half a handful, or in *injections*, one to two ounces of the medicine infused in from two to three ounces of water.

11. *Columbo.*—In habitual diarrahœa of children, and adults. In children, improper nourishment gives rise to a catarrh of the small intestines, or a dysenteric colitis, and from the same cause there is finally developed a state of laxity, and atony of the intestines, manifesting itself by a diarrhœa which is sometimes serous, at others lienteric, which ends in atrophy. This condition does not yield to either calomel, nitrate of silver, or rhubarb; but is sometimes cured by the mild, ferruginous preparations, but especially by the prolonged use of columbo.

℞. *Rad. Calumba*, gr. viij–ʒj; *Aq. Destil.*, ℥j–℥jss; *coque et adde*, *Syrup. Cort. Aurant.*, ℥ss. *S. Take two dessert spoonfuls every hour. Or, thus:* ℞. *Ext. Calumb.*, gr. jvss–gr. vj; *aq. Cannel.*, ℥j–℥jss; *Syr. Cort. Aurant.*, ℥ss. *S To be taken as the preceding mixture.*

12. *Iron.*—M. Hauner employs iron but little. The tincture of the malate of iron, is prescribed in cases of *anamic* conditions, when there is absence of diarrhœa, febrile excitement, and dyspepsia. The syrup of iodide of iron, has proven advantageous in certain cases of scrofula, in cases of extremely feeble children. Thin attenuated children, having a predisposition to pulmonary scrofula, do not well support it, the cough and irritability of the lungs being aggravated by its use.

13. *Jalap.*—One of the best purgatives in cases of children. Children affected with scrofula, ophthalmia, chronic cutaneous eruptions, intestinal worms, slow and irregular digestion, etc.; children of a sluggish habit, large belly, dry skin, slow and indisposed to action, are much more speedily cured when the treatment is begun by an active purgative of jalap and calomel. Neither calomel alone, nor senna, nor aloes, is capable of exciting evacuations as abundant, and as derivative as jalap.

14. *Ipecacuanha.*—As an emetic, its efficacy is well established in many diseases, as *whooping-cough*, *catarrhal fevers*, in cases of very excitable feeble children, predisposed to diarrhœa. In small doses this root renders the greatest service in cases of *bronchial catarrhs*, and spring and autumnal catarrhal affections accompanied with great irritability of these organs, with cough, mucous *rales* in the bronchial tubes, and difficult expectoration, especially when these conditions are complicated with intestinal catarrh.

℞. *Rad. Ipecac.*, gr. ij–gr. jv; *Aq. Bullient.*, ℨjss.–ℨij; *Macera et Cola*; *dein adde Syrup Alb.*, ℨss. *S. Take two dessert spoonfuls every two hours.*

℞. *Rad. Ipecac.*, gr. ij; *Rad. Rhei*, gr. jv; *Aq. Bullientis*, ℨjss; *Macera et Cola*; *adde Syrup Senegæ*, ℨss. *S. Take a dessert spoonful every two hours; or give in powder, from* gr. ⅛ *to* gr. jss, *with sugar and milk every two hours.*

Ipecacuanha is one of the best remedies in *diarrhœas*, frequent with young infants in the hot season. (Summer diarrhœa, dysenteric diarrhœa.) *An infusion of* ℨjss. to ℨij *made with* gr. viij *of Ipecacuanha, with the addition of* ℨss. *of the Syrup of Popy. Two dessert spoonfuls every hour.*

[The best preparation of ipecac is the sacherated. When we use the drug in powder this is the only preparation upon which we can rely. The formula we have given before in the pages of the Journal.—Eds. Journal.]

15. *Creasote.*—Extraordinary means are to be employed only in extraordinary cases, in which the diagnosis is not positive, and in which we are compelled to treat for the more prominent symptoms. To this category belong two cases of *vomiting*, which, resisting every other means applied, yielded to the following portion: ℞. *Creasoti*, *gutt* ij; *aq. Destillat.*, ℨjss. *Syrupi albi.*, ℨss. S. Take two dessert spoonfuls every hour.

16. *Aqua Lauro-cerasi.*—The organism of children is subject to revolutions in cases of irritable subjects, so often badly nourished and badly treated, and to subacute and congestive affections, which not unfrequently manifest themselves in the head, chest, and spinal marrow, with symptoms of nervous irritation, stimulating to a certain extent, cerebral hyperæmia, pneumonia, carditis, myelitis, of which diseases there are only wanting the local pathognomonic signs. The spasmodic pulse, the extreme irritability, the dry skin, the heat, the general agitation, the violent palpitations of the heart, the fatiguing dry cough, under the influence of the *Aq. Laur. Ceras.*, are subjected to a calmative which is unequalled in efficacy by any other medicine. These conditions are moreover frequently met with. This water is still more efficacious in the spasmodic and convulsive cough of children, and in that of consumptives. ℞. *Aq. Laur. Cerasi.*, *gtt.* vj–x–xx; *Syrupi Albi*, ℨj; S. Take half a dessert spoonful every two hours.

17. *Opium, Morphine.*—These agents are not frequently employed by M. Hauner. In the *diarrhœas*, he treats with opium only those which are the mot obstinate; and he uses it in dysentery. He administers it in very minute doses, combined with calomel. ℞. *Pulv. Opii*, gr. 1-30—1-15; *Calomel*, gr. 1-10—one-fifth; *Sacchari Lactis*, gr. viij; *m. ft. pulv.* S. A powder such as this every four hours.

Its use as a calmative is more general; as in *colic pains*, in neuralgias in *insomnia* and *nocturnal cough* of consumptives, (morphine) the cough of the nervous period of the whooping-cough, in non-irritable and phlegmatic children. As an external application, laudanum enters into the composition of numerous collyrii; is used for the dressing of numerous atonic ulcers, in which it favors the formation of healthy granulations; is used in *spina ventosa*, *caries*, etc.

[We cannot too strongly urge upon the attention of the profession the use of the *Liq. morphia comp.*, the formula for its preparation we have also given heretofore.—Eds. Journal.]

18. *Lichen Islandicus.*—Employed as a tonic in mucous affection of the lungs.

19. *Walnut Leaves.*—*Feuilles de Noyer.*—Long experience has proved to M. Hauner that in scrofulous affections, cod-liver oil, so generally recommended, is without effect; that iodine, so very efficacious in certain diseases, the chloruret of barium, antimonials, etc., stand at zero, whilst the walnut leaf, newly dried, shows a decidedly advantageous action. (We do not concur in this condemnation of cod-liver oil; nor as to the infrequency of the good effects of iodine in these affections.—French Translator.) (We have derived excellent results from iodine administered simultaneously with iron.—American Translator.)

The principal indication for the use of walnut leaves, is in cases of torpid, sluggish, and inactive children, in scrofulous ulcers, otorrhœa, chronic, scrofulous, cutaneous eruptions, in atony and engorgements of the lymphatic system. It need scarcely be remarked that the proper hygenic measures ought to be concurrently adopted.

20. *Rhubarb.*—This root will ever find a place in the treatment of children. In young children with affections of the digestive organs, characterized by a tardy expression of the meconium, by icteric symptoms, by non-febrile dyspepsia, etc; in short, conditions known under the names of apepsy, flatulence, acidity, gastricism, caused by feebleness of the digestive organs, the consequence of bad alimentation, are cured readily with one or two dessert spoonfuls, daily, of syrup of chickory with rhubarb. But it must not be given in febrile affections, and in diarrhœa, unless they be purely gastric. At a more advanced age, we increase the activity of the syrup of rhubarb, by adding to it the aqueous tincture of this substance. This combination is prescribed in affections of the liver and spleen, and to close the treatment of intermittent fevers, etc. Infusion:—℞. *Rhei. Contus.*, gr. ijss–gr. viij; *Aq. Bullientis*, ʒjss–ʒij; *macera et cola.* Solution of Extract:—℞. *Ext. Rhei*, gr. ijss–viij; *Aq. Fœniculi*, ʒjss–ʒij; *m. ft. solutio*—this being one of the best stomachics for children artificially nourished.

21. *Senna.*—With children, rhubarb ordinarily supersedes senna; nevertheless, cases occur in which the more energetic action of the latter is to be preferred; and we generally employ it in the form of the Vienna Laxative. Such cases are cerebral inflammations, and congestions in torpid children; obstinate constipation, having its origin in gastric causes, helminthiasis, the onset of gastric fevers, and febrile exanthemata, especially scarlatina and variola, in which it is well to avoid calomel.

22. *Senega.*—In Croup, neither at its onset nor at its acme, has it any favorable action; but it is useful at the period of resotion of the disease, and in consecutive affections; it acts well in the laryngitis which follows measles. In the secretive period of whooping-cough, in chronic bronchitis, with abundant and tenacious accumulation of mucosity, in catarrhal and typhoid pneumonia, and especially in rachitic infants.

The decoction may be prepared by boiling from gr. viij to ʒjv of Senega root, in a sufficient quantity of water, so as to make from ʒjss to ʒij of the decoction, to which may be added ʒss of the sprup of senega, or the medicine may be prescribed in the following form:—℞. Syrup Senega, Syrup Ipecac., Syrup Glycyrrhizæ, aa., ʒss. S. Take two dessert spoonfuls every hour or every two hours.

23. *Valerian Root*, *Carbonate of Magnesia.*—These two agents are much employed, especially in the form of a compound powder, much used in Germany, but almost unknown with us. It is called the children's powder, the pulvis puerorum; for the preparation of which there are various formulæ. The formula preferred by M. Hauner, is according to the manner of Hufeland, except that the oleo-saccharum of fennel is superceded by the sugar of milk. The following is Hufeland's formula:—℞. Magnesiæ Carbonatis, part. xvj; Radicis Rhei, part. vj; Radicis Valerianæ, part. j; Fæniculi Oleo-sacchari, part. viij, m. ft. pulv. Let there be taken, according to the age, what will lay at two or three times on the point of a penknife, daily. However irregular this compound may seem, it is, nevertheless, very valuable; and is very frequently employed. The valerian, by its essential oil, its extractive principle, and its peculiar acid, acts as a tonic on the nervous system; while the magnesia as an antacid, corrects the acridity of the gastric intestinal secretions: the part which is acted by the rhubarb is well known.

24. *Mercury*, principally *Calomel.*—The action of mercury is especially resolvent, promoting the secretions; and hence its frequent employment with infants, in which the diseases of organic life are so numerous. M. Hauner especially prescribes calomel in cases of cerebral affections, in idiopathic meningites, hyperæmia occurring at the period of dentition, in robust children, in convulsions, eclampsia, caused most frequently at that period, by cerebral irritation. In these cases the calomel is given in free doses, say from gr. ⅙, to gr. jss.

The reputation which mercury formerly possessed in cases of hydrocephalic diseases, has vanished before a more correct diagnosis of such cases. The granular affections of the meninges and their result, acute hydrocephalus, are neither cured by calomel, sublimate, nor any other means; and the reported cure in such cases, is founded in error.

Calomel renders important service in all inflammations in which there is a tendency to plastic deposits, and exudations into the internal cavities.

Its favorable action is shown further in intestinal catarrh of children, attended with pain, great sensibility, and distention of the belly, with diarrhœa, sometimes abundant, but more frequently moderate. Secondary cerebral symptoms often complicate such cases, sometimes with somnolence and heaviness of the head, at other times with crying and insomnia, attacking especially children badly nourished and at the same time suffering from dentition. Emaciation and leanness do not contraindicate calomel, though it is important to bear in mind the treatment appropriate to each special case. In affections of the large intestines, characterized by frequent evacuations, with tenesmus and violent, painful efforts, accompanied with cerebral symptoms, often convulsions, even to tetanic cramps, in this true dysentery, calomel is the agent specially indicated; and will alone effect a cure. We give from one-sixth to one-half a grain for a dose, every four or five hours, alone, or associated with feeble doses of opium, say from 1-30 to 1-15 of a grain.

Numerous children have been brought to the hospital suffering from the abuse of the infusion of poppy heads. Insomnia, wasting, dryness of the skin, and trembling, showed themselves, owing to norcosis and hyperæmia of the brain. Calomel in the small doses of from one-sixth to one-half a grain, continued for a long time and aided by cold lotions to the head, has rendered great service in such case.

As an anti-syphilitic, M. Hauner employs the soluble mercury of Hahnemann with the greatest success, while every treatment, as with iodine, sarsaparilla, etc., has had absolutely no effect. In persisting in his plan of treatment for a sufficient length of time, he has secured the permanence of the cure.

25. *Belladonna.*—It always has shown its value in whooping-cough. Nevertheless the last epidemic appeared to be refractory under its use, which M. Hauner explains on the peculiarity of the epidemic, characterized by prolonged catarrhal prodromena, a short spasmodic stage, and a long contiuued terminal cough.

26. *Tartar Emetic.*—This salt in a single dose of from gr. one third to gr. ij, according to the age, has often a wonderful effect in pneumonia, especially the catarrhal, in broncho-pneumonia, in croup, etc. In case of sanguine and robust infants, in marked inflammation, we must employ it after abstraction of blood, and without the latter, in case of feeble and scrofulous children; but diarrhœa and diseases of the digestive apparatus, contraindicate the use of the antimonial. Though its use is less admissible in pure gastric affections; an accumulation of mucosity in the stomach is one of the complications of catarrhal affections of the respiratory organs and in which it may be admissible.

In doses of from 1-30 to one-sixth of a grain, the tartar emetic increases the activity of the secretions and excretions of the skin, lungs, kidneys, intestines, and is indicated in a host of diseases, such as catarrhs, rheumatic, eruptive, and inflammatory fevers, active inflammations, especially of the respiratory apparatus, pulmonary and bronchial catarrhs, and blenorrhagias; affections of the liver and biliary ducts; acute rheumatism, erysipelas, etc.

27. *Cold Water.*—The limits of this article, notwithstanding the importance of the subject, will not permit us to give the details in relation to the use of the agent. M. Hauner refers constantly to articles on the subject previously published, and which we are not in possession of. We can only remark that he employs this agent according to the principles of hydrotherapy in many diseases, and with great success; such as typhoid affections, croup, the dermatoses, scrofulous affections, rickets, diseases of the nervous system, &c.

28. *Cold Baths; Injections.*—These are daily used in a great number of affections, the baths and injections being either simple, medicated, or nutrutive; these latter being composed of froth and the yoke of eggs. In general the indications have nothing special which is not well known; but M. Hauner employs these means much more frequently than most practitioners, and he extols them excessively.

We have already detailed what he has to say in relation to local blood-letting, (he never practices general blood-letting,) in another article.

M. Hauner says nothing of embrocations, sinapisms and blisters, as he rarely employs them.

Although the preceding article deals in many facts which are already measurably known, we have not hesitated to thus give it at some length. It is a summary of the habitual practice of one of our greatest German specialists, who from his position, at the head of a great infantile hospital, is well able to pronounce on the real value of medicines. With this view we give the article entire.

